# Reducing the use of physical restraints in home care: development and feasibility testing of a multicomponent program to support the implementation of a guideline

**DOI:** 10.1186/s12877-020-01946-5

**Published:** 2021-01-25

**Authors:** Sara Vandervelde, Kristien Scheepmans, Koen Milisen, Theo van Achterberg, Ellen Vlaeyen, Johan Flamaing, Bernadette Dierckx de Casterlé

**Affiliations:** 1 KU Leuven, Department of Public Health and Primary Care, Academic Centre for Nursing and Midwifery, Kapucijnenvoer 35 blok d bus 7001, 3000 Leuven, Belgium; 2 Wit-Gele Kruis van Vlaanderen, Nursing Department, Frontispiesstraat 8, bus 1.2, 1000 Brussels, Belgium; 3 University Hospital Leuven, Department of Geriatric Medicine, Herestraat 49, 3000 Leuven, Belgium; 4 KU Leuven, Department of Public Health and Primary Care, Division of Gerontology and Geriatrics, Kapucijnenvoer 35 blok d bus 7001, 3000 Leuven, Belgium

**Keywords:** Home care services, Primary Health Care, Implementation science, Practice Guidelines as Topic, Restraint, physical, Aged

## Abstract

**Background:**

A validated evidence-based guideline was developed to reduce physical restraint use in home care. However, the implementation of guidelines in home care is challenging. Therefore, this study aims to systematically develop and evaluate a multicomponent program for the implementation of the guideline for reducing the use of physical restraints in home care.

**Methods:**

Intervention Mapping was used to develop a multicomponent program. This method contains six steps. Each step comprises several tasks towards the design, implementation and evaluation of an intervention; which is theory and evidence informed, as well as practical. To ensure that the multicomponent program would support the implementation of the guideline in home care, a feasibility study of 8 months was organized in one primary care district in Flanders, Belgium. A concurrent triangulation mixed methods design was used to evaluate the multicomponent program consisting of a knowledge test, focus groups and an online survey.

**Results:**

The Social Cognitive Theory and the Theory of Planned Behavior are the foundations of the multicomponent program. Based on modeling, active learning, guided practice, belief selection and resistance to social pressure, eight practical applications were developed to operationalize these methods. The key components of the program are: the ambassadors for restraint-free home care (*n* = 15), the tutorials, the physical restraint checklist and the flyer. The results of the feasibility study show the necessity to select uniform terminology and definition for physical restraints, to involve all stakeholders from the beginning of the process, to take time for the implementation process, to select competent ambassadors and to collaborate with other home care providers.

**Conclusions:**

The multicomponent program shows promising results. Prior to future use, further research needs to focus on the last two steps of Intervention Mapping (program implementation plan and developing an evaluation plan), to guide implementation on a larger scale and to formally evaluate the effectiveness of the multicomponent program.

## Background

Despite the harmful effects of restraint use on older persons, family caregivers and professional care providers, restraints are still frequently used in home care [1, 2]. A recent systematic review states that, depending on the definition used, the prevalence of restraint use in older persons in home care ranges from 5 to 24.7% [3]. Until recently, no consistent definition of physical restraints could be found in the literature. A Delphi study of Bleijlevens et al. (2016) developed an internationally accepted definition: *“Any action or procedure that prevents a person’s free body movement to a position of choice and/or normal access to his/her body by the use of any method, attached or adjacent to a person’s body that he/she cannot control or remove easily”* [4].

Home care in Flanders is delivered by various professional care providers such as general practitioners (GPs), registered nurses, certified nursing assistants, home health aides, occupational therapists, and physiotherapists. Each professional care provider has an essential role in providing care for people with a care need. This role is in accordance with specific law and regulations of medical and healthcare professions [5]. In Flanders the GPs have a central role in home care. GPs are often key persons in the development of an individual care plan, in close collaboration with specialists and other professional care providers. In the decision-making process for the use of restraints, family, informal caregivers and professional care providers, mainly registered nurses, are involved [1, 6–9]. According to the current legislation, only doctors, registered nurses, certified nursing assistants (if they meet certain conditions such as working in a structured team and under direct supervision of a registered nurse) and informal caregivers (if they meet certain conditions such as training from a nurse or GP, informal caregiver certificate, …) can apply physical restraints when needed [5, 10, 11]. However, literature shows that, in practice, GPs are less involved in the decision-making process and the application of restraints [3]. Also home health nurses in Flanders stated that GPs had no clear role in deciding whether to use restraints [1].

The influence of patient-, nurse- and context related factors make the decision-making process for the use of restraints complex [12]. In particular, the prominent role of the informal caregiver is challenging. A qualitative study reveals that informal caregivers have a dominant role in the use of restraints. This can result in conflicting opinions of restraint use between professional home care providers and informal caregivers [1]. Informal caregivers are significantly less aware of the harmful effects of physical restraints (e.g. bruises, increased dependence, depression) and have a more positive perception of their use [1, 2, 13, 14]. Furthermore, a study concludes that the knowledge of care providers on alternatives for restraint use in home care is limited [6]. The occurrence of conflicting opinions, the lack of awareness of the harmful effects of physical restraint use and limited knowledge among older persons, informal caregivers and professional care providers add to the complexity of the decision-making process in the home care setting and stress the need for a clear policy on restraint use in home care [1, 2]. Therefore, Scheepmans et al. (2016, 2020) developed the first validated evidence-based guideline that aims to increase awareness, knowledge and competences to adequately deal with questions about restraint use in home care [15, 16]. The Belgian Centre of Evidence-Based Medicine (CEBAM) evaluated, validated and approved the scientific quality and reliability of the guideline [17].

However, the development and dissemination of a clinical practice guideline is not sufficient for its integration and routine use in daily practice [18]. A systematic review shows that the rates for adherence to clinical guidelines vary from approximately 20 to 80%, with a median adherence of 34% [19]. The implementation of guidelines in home care organizations entails a complex intervention [20]. Complex interventions, such as multicomponent programs, are interventions that consist of several interacting components, which need change at multiple levels [18, 20–22]. Implementation of a complex intervention requires an exploration of the barriers and facilitators for guideline use, as well as awareness, agreement, adoption and adherence of the adopters during each step of the process [19, 21]. Evidence from residential care settings suggests that using a multicomponent approach involving policy change, leadership and education can reduce the use of physical restraints [23–26]. Yet, the implementation of guidelines is even more challenging in home care [20]. Home care differs from residential care as a result of its particular characteristics like interorganizational structures and team compositions [20]. In home care, where professional care providers enter briefly the personal environment of the older person, they only see the patient for a short amount of time and cannot ensure 24-h coverage and supervision when a person is being restrained [3]. Thus, the specific characteristics of the home care setting make it difficult to translate existing evidence from acute and residential care to the home care setting.

To the best of our knowledge, there is no previous research concerning the implementation of a guideline that aims to reduce physical restraints in home care. Therefore, the overall aim of this study is to systematically develop and evaluate a multicomponent program for the implementation of a guideline for reducing the use of physical restraints in home care.

## Methods

**Fig. 1:**
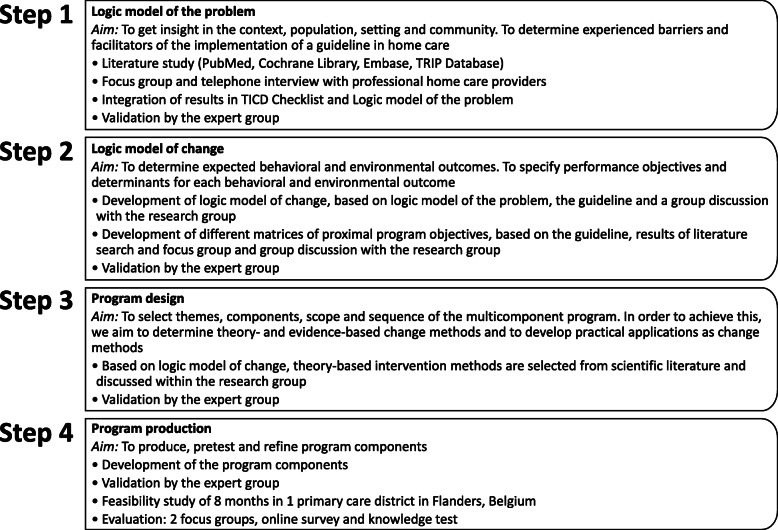
Overview performed steps Intervention Mapping [27]

Intervention Mapping (IM) was used to develop a multicomponent program for supporting the implementation of the guideline for reducing the use of physical restraints in home care [27]. IM provides guidance and tools to ensure that health promotion programs are based on empirical evidence and theories [27]. This mapping approach comprises six steps: (a) producing a logic model of the problem, (b) developing a logic model of change, (c) program design, (d) program production, (e) program implementation plan and (f) developing an evaluation plan. This manuscript describes the operationalization of the first four steps of IM, the last two steps were not performed (Fig. 1) [27].

IM is characterized by the involvement of different stakeholders during each step of the process [27]. An expert group of stakeholders was composed and met six times during the development process (June 2017 – May 2019). It included eleven participants: a GP, a self-employed registered nurse, a registered nurse of a home care nursing organization, two staff members of a home care nursing organization (organization of certified nursing assistants and registered nurses), a staff member of a home care organization (organization of home health aides), a staff member of an organization that represents family caregivers, an occupational therapist, a director of a centre of expertise on dementia, a researcher and a senior academic staff member with expertise in behavioral change theories.

### Step 1: logic model of the problem

The first step of IM consists of a needs assessment. It allows researchers to thoroughly analyze the problem and create a logic model of the problem [27]. A literature search, complemented by a focus group interview with professional home care providers and one telephone interview with a GP, was conducted to get more insight into the context, population, and associated determinants. More information on the methodology of the literature search can be found in additional file 1. The focus group and the telephone interview aimed to obtain feedback from professional home care providers on the identified barriers and facilitators of implementation from the literature search. Participants in the focus group interview were different from the expert group members; and were a GP, a self-employed physiotherapist, a deputy head nurse, a staff member of a home care nursing organization, a registered nurse and a certified nursing assistant. One additional GP who could not attend, participated in a telephone interview. Two researchers moderated the focus group (SV and KS). The interviews followed a topic guide and were recorded (additional file 2). The content of the written text was thematically analyzed, by identifying the key themes (barriers and facilitators) that emerged from the data. The key themes and their underlying meaning were discussed within the research group.

The Integrated Checklist of Determinants of practice (TICD checklist) was used to structure the barriers and facilitators (determinants) based on the main findings of the literature search, focus group and telephone interview [28].

### Step 2: logic model of change

Based on the results of the first step of IM and the content of the clinical practice guideline, the research group developed the logic model of change [15, 16]. This model specifies who and what needs to change to properly manage physical restraint use in home care. In addition, the program outcomes and objectives were specified and the matrices of change objectives were developed. The developed matrices represent detailed change at individual, interpersonal and organizational level and as a consequence the immediate goals of the interventions of the multicomponent program [27]. Next, the proposals of the logic model of change, the program outcomes and the matrices of change objectives were discussed with and evaluated by the expert group. Their feedback was discussed with the research group and integrated in the proposals. The final logic model of change, the program outcomes and the matrices of change were presented to the expert group, prior them granting approval. It concerned an iterative process, in which the research group collaborated with the experts.

### Step 3: program design

The next step was to select theory- and evidence-based methods, which could be effective in achieving the main objectives. Bartholomew et al. (2016) give an overview of theory and evidence-based methods that match certain determinants, which can be translated into practical applications [27]. The theory-based intervention methods and evidence-based intervention applications were selected from this overview [27, 29]. Based on the creativity, relevance, potential effectiveness and feasibility of the practical intervention applications, the expert group decided on a final selection of methods and applications.

### Step 4: producing and testing program components

The fourth step of IM consists of producing the practical program components and testing and evaluating the program with the target population [27]. Practical components were developed by the research group, with iterative feedback from the expert group. The multicomponent program was tested and evaluated in a feasibility study.

#### Feasibility study

The feasibility study was performed from February 2018 until October 2018 in one of the 59 primary care districts in Flanders (Belgium). This care district contains six municipalities or care regions with a total of 103.225 inhabitants [30]. The Department of Welfare, Public Health and Family (Flanders) developed a website that provides an overview of professional home care providers and organizations in Flanders and Brussels. Based on this website, the researchers made an overview of active professional home care providers and organizations in the selected care district. In addition, the researchers asked a local multidisciplinary network (network of GPs and other professional home care providers) of the care district to send an e-mail invitation to their network members. All professional home care providers (i.e. nurses, certified nursing assistants, GPs, physiotherapists, and occupational therapists) and organizations (i.e. home nursing organizations, home care nursing organizations, home care organizations, senior and community centers, organizations that provide assisted living) of the selected district were contacted by email and were asked to participate. All the interested professional home care providers and organizations received information and an informed consent form. At the start of the feasibility study an information session was organized, during which the guideline and multicomponent program were presented. After this session, the professional home care providers and organizations were asked to give their informed consent for their participation. Professional home care providers who were actively engaged in the program (*n* = 15), received a training for becoming an ambassador for restraint-free home care and were stepwise exposed to all the components of the program through emails and a website. During the feasibility study, the newly trained ambassadors received two peer coaching sessions and one telephone call for process guidance.

#### Evaluation of the multicomponent program

A concurrent triangulation mixed methods design was used to evaluate the developed multicomponent program [31]. In this study, quantitative and qualitative data were collected simultaneously, but analyzed separately. The different results were merged during interpretation [31]. The evaluation consisted of a knowledge test for the ambassadors and all professional home care providers from the participating organizations. The knowledge test was not externally validated. However, the research group cautiously developed and evaluated the knowledge test based on the content of the guideline. This test had 0 as the minimum score and 31 as the maximum score. Furthermore, two focus group interviews and an online survey were held with the ambassadors to perform a process evaluation. They evaluated the different components of the multicomponent program and the feasibility of implementing a guideline of physical restraint use in home care. More information on the methodology of the knowledge test, online survey and focus groups can be found in additional file 3 and the topic guide of the focus group interviews can be found in additional file 4.

The final results of the knowledge test, the focus groups and survey were presented to the expert group and also to the ambassadors for restraint-free home care. They were asked if the results were accurate and in line with their experiences (i.e. member checking).

## Results

### Step 1: logic model of the problem

**Fig. 2.**
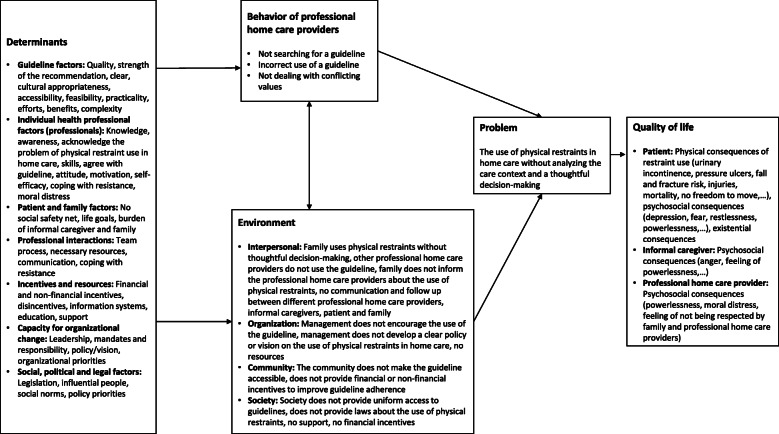
Logic model of the problem

The results of the needs assessment can be found in the logic model of the problem (Fig. 2). ‘Quality of life’ (QoL) is the ultimate outcome and formed the starting point of the model. By placing the focus on QoL, the researchers were stimulated to think backwards through the logic model of the problem to identify the problem, the behavior of professional home care providers, the environment and the determinants influencing the behavior and environment. The logic model of the problem helped the researchers to plan, implement and evaluate the program with the end in mind [27].

#### Quality of life

Physical restraint use has an impact on QoL of the patient, the informal caregiver and the professional home care providers. The patient can experience physical (e.g. urinary incontinence, pressure ulcers, falls, …), psychosocial (e.g. depression, fear, …) and existential consequences [32, 33]. The use of restraints also comes with negative psychosocial consequences for the informal caregiver (e.g. anger, powerlessness) and professional home care providers (e.g. frustration, moral distress) [1, 14].

#### Problem

Due to demographic, epidemiological, social and cultural trends, there is a growing number of older persons living at home [34]. These older persons often have chronic conditions, which are associated with restraint use. Consequently, professional home care providers are increasingly confronted with the use of physical restraints [9, 33]. Findings from the needs assessment indicate that currently physical restraints are being used without analyzing the care context and thoughtful decision-making [1, 2, 6].

#### Behavior of professional home care providers and environmental factors

Based on the findings of the literature search, the focus group interview and the expert group meeting, different behavioral and environmental factors leading to a lack of thoughtful decision-making were identified. Not searching for a validated guideline, incorrect use of the guideline and not dealing with conflicting values are behavioral factors at the level of home care providers. The environmental factors are classified into four levels; interpersonal, organization, community and society. The most important environmental factors at interpersonal level are the use of physical restraints by family without thoughtful decision-making and the lack of communication between home care providers, informal caregivers, family and patient. At the organization level, a lack of encouragement from management to use guidelines is a crucial factor. No access to guidelines for all professional home care providers and the absence of financial and non-financial incentives to improve guideline adherence are the most commonly mentioned environmental factors on community and society level.

#### Determinants

The most frequently-mentioned determinants are: the feasibility and practicality of the guideline (guideline factors); the knowledge, motivation and awareness of the professional home care providers (individual health professional factors); the burden of informal caregivers and family, no social safety net and the alignment with the life goals of the older person (patient and family factors); communication between home care providers (professional interactions); financial and non-financial incentives (incentives and resources); leadership and organizational priorities (capacity for organizational change); legislation and policy priorities (social, political and legal factors).

### Step 2: logic model of change

#### Logic model of change

**Fig. 3:**
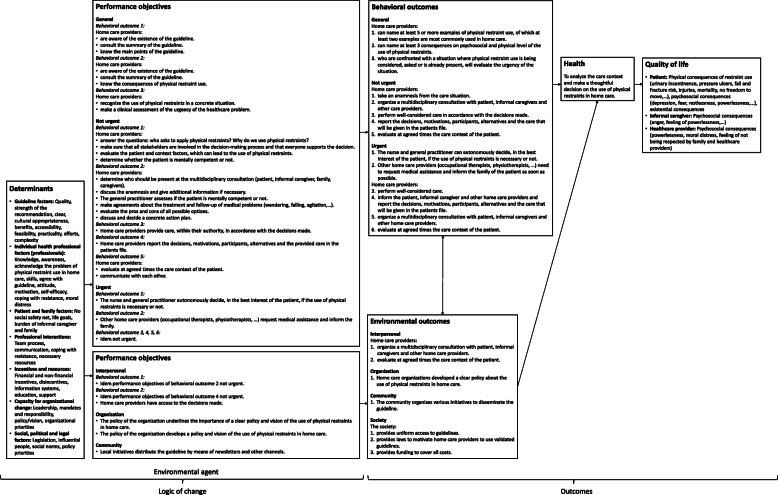
Logic model of change

Figure 3 shows the logic model of change. The overall health objective is that home care providers analyze the care context and make a thoughtful decision on the use of physical restraints in home care. The logic model contains 14 behavioral change outcomes. Additionally, the logic model has seven environmental outcomes at interpersonal, organization, community and society level. The main behavioral outcomes focus on the awareness of the problem of physical restraint use, the knowledge of the guideline and the need for clear communication and collaboration between different care providers, patient, informal caregiver and family. The environmental outcomes mainly focus on a clear vision or policy regarding physical restraint use in home care organizations. Above that, the dissemination and the accessibility of the guideline are taken into account. For each behavioral and environmental outcome different performance objectives are formulated (Fig. 3).

#### Matrices of change objectives

For each behavioral and environmental outcome a matrix of change objectives was developed. The matrices were constructed by combining performance objectives with determinants and defining specific change objectives. These matrices form a concrete pathway for behavioral and environmental changes [27]. An example of a matrix of change objectives can be found in Table 1.

**Table 1:** Matrix of change objectives (Example)

Behavioral outcome 3 (general): Home care providers who are confronted with a situation where physical restraint use is being considered, asked or is already present, will evaluate the urgency of the situation										
	Complexity of the guideline	Knowledge	Awareness	Skills	Motivation	Self-efficacy	Burden of informal caregiver and family	Presence of family	Communication	Education
**PO1**^a^**: Recognize the use of physical restraints in a concrete situation**	The term ‘physical restraints’ needs to be clearly defined in the guideline.	Know and understand the term physical restraints.	Are aware of the different types of restraint use.		Is motivated to recognize physical restraints in home care.	Say they can name five examples of physical restraints.				Have received education on the content of the guideline.
**PO2**^a^**: Make a clinical assessment of the urgency of the healthcare problem**		Have knowledge of the consequences of physical restraint use on patients, informal caregivers, family and home care providers.	Are aware of the consequences of physical restraint use on patients, informal caregivers, family and home care providers.	Can assess the risks of the patient, informal caregiver, family and home care providers.	Want to improve the quality of care and quality of life. Want to avoid serious consequences for patient, informal caregiver, family and home care providers.	Say they can make a clinical assessment of the urgency of the healthcare problem.	Know the patient and context factors associated with physical restraint use. Have a trust relationship with patient, informal caregiver and family.	Know the social support of the patient. Know how often family is visiting the patient.	Communicate with patient, informal caregiver, family and other home care providers.	Have received education on the consequences and the impact of physical restraint use on patients, informal caregivers, family and home care providers.

^a^(PO) Performance objectives

### Step 3: program design

In the third step of IM, the research group and expert group of stakeholders selected theory- and evidence-based methods to influence the determinants identified in the logic model of change and the different matrices of change objectives. The main theories behind the multicomponent program are the ‘Social Cognitive Theory’ and the ‘Theory of Planned Behavior’ [27, 35]. From the Social Cognitive Theory the researchers selected ‘modeling’, ‘active learning’ and ‘guided practice’ as evidence-based methods to influence the identified determinants. With modeling we aim to provide the professional home care providers an appropriate role-model, more specifically an ambassador for restraint-free home care. If the home care providers see and observe successful demonstration of behavior by a role model, they can reproduce the same behavior. The ambassadors receive a one-day training, where the trainers use the method ‘active learning’, learning based on goal-driven and activity-based experience. In addition, this training consists of ‘guided practice’. The ambassadors rehearse and repeat behavior various times by means of role play. After the role play, peers discuss the behavior and give feedback. The main evidence-based methods selected from the Theory of Planned Behavior are ‘belief selection’ and ‘resistance to social pressure’. The strategy behind the method ‘belief selection’ is to use messages designed to strengthen positive beliefs and weaken negative beliefs about physical restraint use in home care. With this strategy in mind the researchers developed a flyer and a promo video. For the method ‘resistance to social pressure’, the ambassadors receive a training and peer coaching sessions to build skills for resistance to social pressure. Table 2 gives an overview of all the selected theories, methods, implementation strategies and the practical components of the program.

**Table 2:** Implementation strategies and related behaviour change methods [27, 35]

Determinants	Theory	Method	Strategy	Components
Dissemination and accessibility of the guideline	Theories of Social Networks and Social Support Theory of Learning	Mobilizing social networks Repeated exposure	Mobilizing social networks: Ask social networks to disseminate the guideline Repeated exposure: Make a stimulus repeatedly accessible	Website Social media Summary of the guideline
Knowledge	Social Cognitive Theory Trans-Theoretical Model	Active Learning Tailoring Individualization	Active learning: Learning based on goal-driven and activity-based experience Tailoring: Matching the components of the program to characteristics of the participants Individualization: Providing the opportunity for ambassadors to have personal questions answered	Ambassador for restraint-free home care Peer coaching sessions Summary of the guideline Physical restraints checklist Flyer Tutorials Telephone follow-up
Awareness	Theory of Planned Behavior Health Belief Model Theories of Social Networks and Social Support	Belief selection Consciousness raising Mobilizing social networks	Belief selection: Using messages designed to strengthen positive beliefs and weaken negative beliefs about physical restraint use in home care Consciousness raising: Providing information, feedback on a problem/situation Mobilizing social networks: Ask social networks to disseminate the guideline	Promo video Flyer Peer coaching sessions Social media
Attitude and motivation	Theory of Learning Theory of Planned Behavior	Repeated exposure Belief selection	Repeated exposure: Making a stimulus repeatedly accessible Belief selection: Using messages designed to strengthen positive beliefs and weaken negative beliefs about physical restraint use in home care	Stepwise exposure to the different components of the program by email and social media Promo video
Self-efficacy	Social Cognitive Theory	Guided practice	Guided practice: Rehearse and repeat behavior various times, discuss and give feedback	Ambassador for restraint-free home care Peer coaching sessions
Communication	Models of Community Organization	Community development	Community development: Community in which power is shared and members engage together	Creating a network of ambassadors for restraint-free home care
Qualified staff	Social Cognitive Theory Theory of Planned Behavior	Modeling Resistance to social pressure	Modeling: Providing an appropriate role-model Resistance to social pressure: Building skills for resistance to social pressure	Ambassador for restraint-free home care Peer coaching sessions

**Fig. 4:**
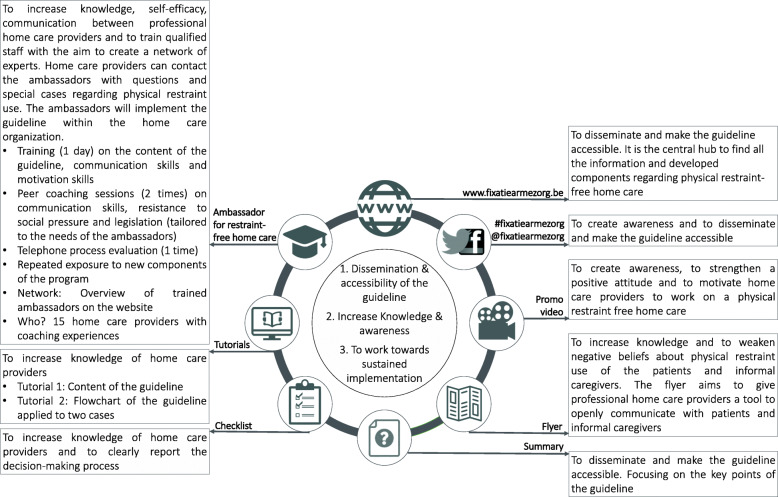
Different components of the multicomponent program

The developed multicomponent program has three main objectives: [1] to make the guideline more accessible and to disseminate it, [2] to increase awareness and knowledge of the problem of physical restraint use in home care, and [3] to work towards sustained implementation. Based on the theory- and evidence-based methods, the research group and expert group of stakeholder selected and designed eight practical applications to operationalize those methods; i.e. a website, social media, promo video, flyer, summary of the guideline, physical restraints checklist, tutorials and ambassadors for restraint-free home care. More information on the different components of the program can be found in Fig. 4.

### Step 4: producing and testing of program components

In step 4 of IM the multicomponent program (Fig. 4) was tested for 8 months (February – October 2018) in one primary care district in Flanders, Belgium. In total, 15 professional home care providers received a training for becoming an ambassador for restraint-free home care. One professional home care provider was a self-employed occupational therapist, the other ambassadors worked for various home care organizations, being: home care nursing organizations (*n* = 8) home care organizations (organization of home health aides; *n* = 3), a senior and community center (*n* = 1), an organization that provides assisted living facilities (*n* = 1) and an adult day care center (*n* = 1).

#### Knowledge

After 8 months of testing the multicomponent program, a knowledge test was completed by 73 home care providers of the participating organizations, of which 13 were ambassadors and 60 were non-ambassadors (Table 3). The participants were mainly women (*n* = 71), with a mean age of 41.5 ± 10.6 years. The majority of the participants were certified nursing assistants (*n* = 23), home health aides (*n* = 22) and registered nurses (*n* = 20).

**Table 3:** Demographic variables participants of the knowledge test

	Non-ambassadors (***n*** = 60) (%)	Ambassadors (***n*** = 13) (%)
**Sex**		
Female	58 (97)	13 (100)
Male	2 (3)	
**Age**		
≤ 30 years	9 (15)	1 (8)
31–40 years	18 (30)	2 (15)
41–50 years	12 (20)	2 (15)
> 50 years	9 (15)	8 (62)
Unknown	12(20)	
**Discipline**		
Certified nursing assistant^a^	23 (38)	
Registered nurse	11 (19)	9 (69)
Occupational Therapist		3 (23)
Home health aide^b^	22 (37)	
Social worker	2 (3)	
Unknown	2 (3)	1 (8)
**Team manager**		
Yes	6 (10)	6 (46)
No	54 (90)	7 (54)
**Highest level of education**		
Secondary education degree	39 (65)	
Certificate of advanced vocational education	13 (22)	3 (23)
Bachelor’s degree	5 (8)	10 (77)
Bachelor’s after bachelor’s degree	2 (3)	
Master degree	1 (2)	
**Working experience in home care**		
< 5 years	13 (22)	3 (23)
5–10 years	14 (23)	1 (8)
11–20 years	25 (42)	5 (38)
> 20 years	8 (13)	4 (31)

^a^Certified nursing assistants: A certified nursing assistant supports and helps patients with activities of daily living and healthcare needs, under supervision of a Registered Nurse (RN) [10]

^b^Home health aides: Home health aides assist patients with activities of daily living and provide basic routine care [36]

**Fig. 5:**
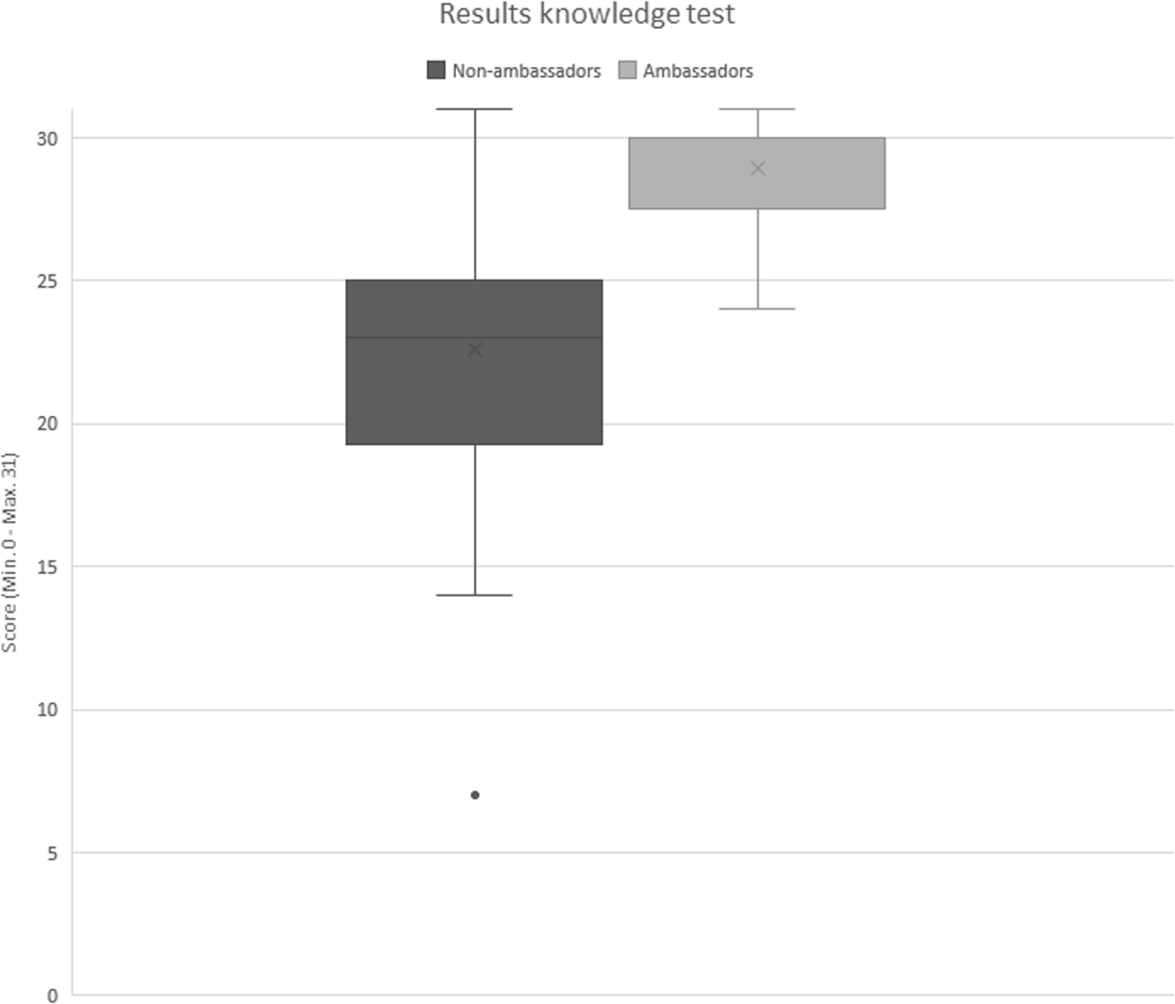
Results of the knowledge test for non-ambassadors (*n* = 60) and ambassadors (*n* = 13)

The ambassadors scored noticeably higher on the knowledge test (mean score = 28.9 ± 1.98) (than the non-ambassadors (mean score = 22.6 ± 4.36) (Fig. 5). The non-ambassadors scored less on questions about the alternatives for physical restraints and the legislative framework for physical restraint use in home care.

#### Process evaluation

A process evaluation was performed after 8 months of delivering the multicomponent program. Ten out of fifteen ambassadors participated in the online survey (results in additional file 5) and nine out of fifteen ambassadors participated in the focus groups. The results of the process evaluation are described in two main topics: [1] the evaluation of the multicomponent program and [2] the perceived barriers.

1. Multicomponent program

The results of the focus group interviews and the online survey show that the ambassadors acknowledged and appreciated the added value of various components of the program. Several components increased their knowledge and awareness of the problem of physical restraint use.

*“The multicomponent program is a valuable framework to support us to achieve a physical restraint-free home care. Otherwise it wasn’t feasible for us.”*

*“The multicomponent program was very important for awareness. It was the first step to work on a policy within our organization”.*

The results of the process evaluation show that not all components were evaluated equally positive. During the interpretation of the results of the survey and the focus group interviews, the researchers could identify key components, valuable components and optional components. The key components are those components that are evaluated as the most crucial and useful components of the program. The valuable components are evaluated as useful and helpful, but study results indicate that they are not seen as the most essential components of the multicomponent program. The optional components are deemed valuable to particular professional home care providers, but for the ambassadors these components are less helpful and not appealing.

#### Key components of the multicomponent program

Based on the results of the online survey and focus group interviews, the ambassadors for restraint-free home care, the tutorials, the physical restraint checklist and the flyer are defined by the researchers as the key components of the multicomponent program. According to the majority of the ambassadors, the training for becoming an ambassador restraint-free home care ensured that they could support their colleagues. All the ambassadors found that this training provided them with the necessary skills to give feedback to colleagues. Nine ambassadors stated that the training helped them to deal with resistance from colleagues. In addition, both the results of the online survey and the focus group interviews showed that peer coaching sessions and the telephone follow-up by the researcher continuously motivated and stimulated them to work on a physical restraint-free home care.

*“The peer coaching sessions put the spark back in our work towards physical restraint-free home care”.*

*“In the telephone follow-up, you ask questions “what are you doing, what is your progress?” And then we start to think, how are we going to do it? “.*

The two peer coaching sessions helped the majority of the ambassadors to understand the legislation relevant to physical restraint use in Belgium and provided them with more insight into the different alternatives for restraint use. In the focus group interviews the ambassadors stipulated that they received information on the alternatives for physical restraint use, but there is still a need to define and provide alternatives.

*“Legislation was very important and the alternatives were also important.”*

*“Can you develop something on the alternatives for physical restraints? Where can you get it? What is the price? Is it covered by the insurance company? What are useful tools?”*

Participants indicated in the focus group interviews that they used the flyer to communicate with patients, families and caregivers, because it was compact, brief and concise.

*“The flyer was also important. Because how do you go to the informal caregiver and discuss the use of physical restraints? The flyer is a useful tool.”*

Also the results of the focus group interview and online survey show that the physical restraint checklist was perceived as a helpful tool, since it matched their daily working method and it supported the majority in documenting the care situation and the decision-making process. Other key components of the program are the tutorials on the guideline and on the flowchart. In the focus group interviews the ambassadors evaluated the tutorials as useful and recognizable and it continuously motivated and stimulated them to work on a physical restraint-free home care.

*“The tutorials are very useful, the guideline is explained in an amusing way, and the cases appeal to the imagination.”*

In the online survey, eight ambassadors found that the tutorial on the guideline raised awareness, supported and motivated them to use the guideline. All the ambassadors that have seen the tutorial on the flowchart believed that the tutorial supported home care providers in their daily practice, motivated them and clarified the use of the flowchart.

#### Valuable components of the multicomponent program

The results of the online survey and focus group interviews show that the website and the promo video were seen as valuable components. All the ambassadors evaluated the website as logical and clear and it raised their awareness. Nine ambassadors indicated that the website supported them in their daily practice.

*“I think the website is very important. We will also use it in the training of our professional home care providers.”*

The promo video was well evaluated by the majority of the ambassadors, it increased awareness and it motivated people to work on a physical restraint-free home care. The ambassadors found that due to their education and experience, the professional home care providers already knew the content covered by the promo video. Therefore, the promo video could be more useful for the patient, family and informal caregivers.

*“The promo video is for* a broader audience*, who do not know anything about it. It is important and convenient. If people already know the content, it is difficult to keep their attention.”*

#### Optional components of the multicomponent program

The social media pages and the summary of the guideline are less well evaluated by the ambassadors. The majority of the ambassadors found the social media pages (Facebook and Twitter) less helpful and not appealing. Half of the ambassadors did not visit the social media pages.

*“Social media, I am not into social media. I have not been interested in social media and it does not appeal to me at all, maybe for young people.”*

The summary of the guideline aimed to support the professional home care providers in the analysis of the care situation and the decision-making process. In the focus group interviews, the ambassadors indicated that the summary of the guideline was not useful and too complex. A minority of the ambassadors used the summary monthly.

*“The flowchart, part of the summary of the guideline, is too complex to use especially for home health aides. We have made adaptations.”*

2. Perceived barriers to the implementation of the guideline

Several perceived barriers to the implementation of the guideline are identified from the focus group interviews. The ambassadors experienced that, in practice, the **term ‘physical restraints’** is being interpreted too narrow; only the most extreme and least acceptable methods (e.g. ropes, belts) were taken into account. Due to the fact that ‘physical restraints’ has a negative connotation and home care providers were not aware of the full meaning of this term, it resulted in limited recognition of the problem. So, the narrow interpretation of ‘physical restraints’ by the ambassadors and other home care providers formed a barrier to fully exploit the added value of the multicomponent program for the implementation of the guideline. The ambassadors found it important to think about a more suitable and uniform terminology and a clear definition for physical restraints, so that confusion could be avoided.

*“Locking the door or room, people don’t see this as physical restraints … Also if you prevent someone from going upstairs. Not everyone sees this as physical restraints.”*

The ambassadors found the **fragmented approach in home care** a challenge when trying to implement a guideline. They found it difficult to involve and collaborate with different care providers such as self-employed nurses, GPs and physiotherapists. The ambassadors indicated that a common vision, general agreements and uniform documents are important to facilitate this collaboration.

*“We want to do it, but if the other care providers are not part of the story, we will remain in the physical restraints circle.”*

The **legislation on physical restraint use** was experienced as an important barrier to implement the guideline in home care. In Belgium, only doctors, nurses, certified nursing assistants (if they meet certain conditions such as working in a structured team and under direct supervision of a registered nurse) and informal caregivers (if they meet certain conditions such as training from a nurse or GP, informal caregiver certificate, …) can apply physical restraints [5, 10, 11]. The fact that an informal caregiver can be allowed to apply physical restraints and that certain home care providers (e.g. occupational therapists, home health aides, and physiotherapists) cannot, influenced the self-image and self-confidence of these care providers. In addition, the ambassadors indicated that the current legislation is restrictive for some professional home care providers.

*“The legislation is very restrictive for home care. If you apply it strictly, we will give the home health aides the feeling that they are unneeded.”*

*“We have been very careful and have not explained the content of the guideline explicitly to the home health aides.”*

The ambassadors experienced a **lack of time** for facilitating the implementation as an important barrier. The entire process requires effort and time. The implementation process must be well thought out and prepared, before the actual start. With an implementation period of only 8 months, all ambassadors perceived the feasibility study as too short.

*“It is such a short period of time to realize it. And it takes time to become more aware and to let everything settle. And for an organization you have far too little time to implement something. You solely have time to create awareness.”*

Another challenge experienced by the ambassadors was the **lack of involvement and support of their managers**. The ambassadors found it necessary that managers set priorities and develop a common vision and implementation plan related to the use of physical restraints. Not all of the ambassadors had the **organizational power** to implement a guideline on physical restraint use within their organization, which formed a barrier for the implementation process.

*“The management is not yet on board. We need to involve them in order to implement it. We are now working on a vision or policy. That has been the bottleneck, to continue and have a complete concept. Everyone has to go along, including managers. It must be supported by the organization and the management.”*

## Discussion

This study developed and evaluated a complex intervention to support the implementation of a guideline for reducing the use of physical restraints in home care. Modeling, active learning, guided practice, belief selection and resistance to social pressure are the evidence-based methods used to select the eight practical applications. The developed multicomponent program has three main objectives: to disseminate and make the guideline more accessible, to increase awareness and knowledge of the problem of physical restraint use and to work towards sustained implementation. This multicomponent program consists of eight practical components (website, social media, promo video, flyer, summary of the guideline, physical restraints checklist, tutorials and ambassador restraint-free home care). The guideline for reducing the use of physical restraints in home care is not openly accessible and therefore it is not part of the developed multicomponent program [15, 16]. It could be assumed that this might form a barrier to using the guideline. For this reason, a summary of the guideline was developed. This summary contains the key points of the guideline and it also consists of the flowchart that guides professional home care providers through the decision-making process. In addition, the content of the guideline was extensively explained and discussed in the training to become an ambassador for restraint-free home care. The ambassadors for restraint-free home care received a free copy of the guideline.

The results show that the multicomponent program is useful for implementing the guideline in home care. The ambassadors positively received, experienced and evaluated various components of the program. Components that were recognizable, compact, brief and concise, such as the physical restraints checklist, tutorials and flyer, were best evaluated. The ambassadors indicated that due to the combination of the different components of the program their knowledge, skills and awareness of the problem of physical restraint use in home care had increased. Especially the tutorials and the training to become an ambassador restraint-free home care, including peer coaching sessions and telephone follow-up, are considered essential for the program. The website and promo video are valuable, but are not the essential components of the program. In the focus group interviews the ambassadors did not put as much emphasis on the website and the promo video in comparison to the key components. Optional components of the multicomponent program are the social media pages and the summary of the guideline. The ambassadors thought the social media pages were less appealing and saw the summary of the guideline, more in particular the flowchart, as too complex.

This study also highlights barriers to the implementation of the guideline. First, the term ‘physical restraints’ is interpreted too narrowly. For this reason, it forms a barrier to fully exploiting the added value of the multicomponent program for the implementation of the guideline. Some ambassadors indicated that professional home care providers were not aware of the broad definition of physical restraints as used in the guideline [15, 16]. Only the extremes, such as belts and ropes, were taken into consideration, resulting in a limited recognition of the problem. From a literature search, Bleijlevens et al. (2016) identified 34 different definitions of physical restraints [4]. The ambiguity about the term ‘physical restraints’ is well known [37, 38]. The results from our study further emphasize the need to search for a uniform term that describes the full scope. Second, the fragmented approach in home care is also a challenge. A lack of common vision, general agreements and uniform documents impedes the implementation process. A systematic review of reviews reveals that collaboration and good coordination between the different stakeholders and organizations is important for implementation. Shared decision-making, non-hierarchical relationships, mutual respect, trust and open communication are essential characteristics of good collaboration [20]. Another important barrier is the lack of involvement and support of managers. Literature also underlines that support and commitment from managers who reaffirm the importance of change are important facilitators for successful implementation in home care [20, 39–43]. In addition, the ambassadors felt that they did not have the organizational power to carry out this change project within their organization. Earlier studies show that the absence of staff with the right competences or expertise impedes implementation [20, 39, 40, 42–48]. For this reason, the research group formulated desirable competences (e.g. coaching skills, leadership). The participating organizations selected suitable candidates for becoming an ambassador for restraint-free home care. However, we did not verify if the candidates actually had these competences and organizational power to carry out this change project. It is possible that not all of the selected candidates had the right competences (e.g. leadership, coaching skills) to facilitate the implementation of the guideline. Another barrier is the lack of time for facilitating the implementation of the guideline. Due to the relatively short implementation period, the ambassadors felt they could only raise awareness of the problem of physical restraint use in home care. Indeed, literature shows that a lack of time for planning and implementing new interventions or procedures is a barrier. The organizational readiness (e.g. staff, training, strategic planning, resources) and the extent to which a new intervention fits in the current workflow influence the implementation process [20, 37, 42, 43]. Lastly, various ambassadors perceived the current legislation regarding the use of physical restraints in home care as an important barrier. Currently, the legislation regarding physical restraints use in Belgium is not clear. In Belgium, not all professional care providers can apply physical restraints when needed. The problem is that the current legislation regarding physical restraints is restrictive. For example, when a person is restrained and a professional home care provider, who is not allowed to apply restrains, is taking care of this person, the professional home care provider needs to contact a doctor or a registered nurse to reapply the restrains when needed. However, in home care a doctor or a registered nurse is not always available, which could mean that the use of restraints is either discontinued or that restraints are applied by persons who are neither authorized nor prepared for this. Therefore, the current legislation makes it difficult to perform integrated care and for this reason, it is complex to cooperate with different professional home care providers [5, 10, 11]. Literature reveals that the presence of an appropriate legislative framework is a powerful activator; while a lack of clarity about roles, responsibilities and tasks within the implementation process acts as important barrier. In addition, concerns about less autonomy, trust and independence impede the implementation of change [20, 37].

This study uses Intervention Mapping in line with the widely used and cited United Kingdom Medical Research Council (MRC) framework for developing and evaluating complex interventions. The MRC framework provides a useful general approach to systematically design and evaluate complex interventions. The key elements of the development and evaluation process are: development, feasibility and piloting, evaluation and implementation [18]. In addition, this study uses Intervention mapping, which provides a systematic and logic process for intervention development, implementation and evaluation in accordance to the criteria of the MRC framework [27]. Yet, intervention mapping provides researchers more detailed and specific guidance during the development of the intervention [49–51]. Therefore, an important strength of this study is the use of Intervention Mapping in the systematic development of the multicomponent program [27]. By using this mapping approach, we applied four perspectives during all steps of the development process. With the (a) participation perspective, we intended to involve the target group and program implementers. (b) The multi-theory perspective stimulated us to approach real-life problems with multiple theories. (c) The systems perspective indicated that interventions need to be seen as part of a system, with interacting factors. (d) Finally, with the social and ecological perspective, we took the impact of the social and ecological conditions on behaviour into account. The developed multicomponent program includes clear objectives, methodologies and relates to behavioral change theories [27, 52]. Another strength of this study is that we performed a process evaluation of the multicomponent program with the intended program adopters. A process evaluation is an essential part of designing and testing a complex intervention, such as a multicomponent program for the implementation of a guideline [53]. The feasibility study is useful for getting a sense of how care providers perceived and evaluated the different components of the program [18, 21]. In addition, the process evaluation gave us more insight in the contextual factors (e.g. perceived barriers and facilitators), the implementation process (e.g. the use of the different components of the program) and the mechanisms of impact (e.g. participants’ responses to the different components) [53]. These results can be used to optimize the multicomponent program.

Nonetheless, it is important to note the limitations of this study. The first limitation is the limited involvement of management. A change requires time, resources and sufficient support. Therefore, the involvement of this group is already crucial during the development phase and should be strengthened in future efforts. Another limitation is that patients, informal caregivers and self-employed home care providers are insufficiently represented in the development phase of the study. Various initiatives were taken to involve these groups; but this proved to be difficult. A possible explanation for their absence, is that given the sensitivity of this topic and the negative connotation of the term ‘physical restraint use’, no patients, informal caregivers or self-employed home care providers were willing to participate. There are also some limitations of the feasibility study. First, the knowledge test was cautiously constructed based on the content of the guideline and evaluated by the researchers of the research group. Yet, the knowledge test was not externally validated, and therefore the results for this knowledge test need to be interpreted with some caution. Second, only two thirds of the ambassadors participated in the online survey (*n* = 10) and the focus groups (*n* = 9). Not all of the trained ambassadors evaluated the multicomponent program. A possible reason for not evaluating the multicomponent program can be the limited duration of the feasibility study (8 months). The ambassadors were still working towards increasing awareness. Not all the ambassadors had the time to use the different components of the program. It can be assumed that we performed the evaluation too early in the process. For this reason it is important to interpret the results of the process evaluation with caution. The management is also insufficiently involved in the feasibility study. This could explain why the ambassadors did not experience support from the management of the organization. Lastly, we let the participating organizations select the suitable candidates for becoming an ambassador for restraint-free home care. The findings of this study emphasize the necessity to carefully select the ambassadors based on strict competences (e.g. motivation, coaching skills, experience with change projects, leadership).

Prior to further implementation, future research needs to focus on the fifth and sixth step of IM. An integral plan for wider implementation needs to be developed (step 5 of IM – Program implementation plan). In addition, it is important to determine the effects of the multicomponent program on the attitudes, self-efficacy, knowledge and skills of the professional home care providers. Furthermore, we need to gain more insight into the implementation outcomes (reach, dose, fidelity) and the effect of the multicomponent program on the use of the guideline for physical restraint use in home care (e.g. cluster randomized controlled trial, hybrid designs) (step 6 of IM – Evaluation plan).

## Conclusions

We can conclude that the multicomponent program shows promising results for implementing the guideline for reducing the use of restraints in home care. The multicomponent program is necessary, yet not fully sufficient to guide the full implementation of this guideline. Prior to further implementation, research is still necessary and needs to focus on larger scale implementation and evaluation of the effect of the multicomponent program. For future implementation it is important to involve all stakeholders from the beginning of the implementation process, use uniform terminology and a uniform definition for physical restraints, select competent ambassadors, assure buy-in of the management and facilitate collaboration between different home care providers.

## Supplementary Information

**Additional file 1.** Methodology literature search.

**Additional file 2.** Topic guide focus group interview IM step 1.

**Additional file 3.** Methodology evaluation multicomponent program (knowledge test, online survey and two focus groups).

**Additional file 4.** Topic guide focus group interviews IM step 4.

**Additional file 5.** Results survey evaluation multicomponent program.

## Data Availability

All data generated or analyzed during this study are included in this published article and its supplementary information files.

## Abbreviations

IM: Intervention Mapping
GP: General Practitioner
QoL: Quality of life
