# Supplementary material for: Reducing the use of physical restraints in home care: development and feasibility testing of a multicomponent program to support the implementation of a guideline
Source: BMC Geriatr. 2021 Jan 25;21:77. doi: 10.1186/s12877-020-01946-5 (PMC7831193; doi:10.1186/s12877-020-01946-5)
Supplement: Supplementary file 1 — Additional file 1. Methodology literature search. [file 12877_2020_1946_MOESM1_ESM.docx]

**Additional file 1: Methodology literature search**

A search in PubMed, Cochrane Library, Embase and TRIP Database was performed for the period from July 2017 until August of 2017. A search string was developed in collaboration with a biomedical library expert. Index and free text were combined with Boolean operators, depending on the database.

Search terms were "Home Care Services", "Domiciliary Care", "home care", "home care agencies", "home care agency", "home health agencies", "home health care agency", "home health care agencies", "Primary Health Care", "Primary Care", "Primary Care Nursing”, "Home Nursing", "community care", “community health”, “community health service”, ”community health care”, ”community healthcare”, "nurses, community health", "Community Health Nurse", "Community Health Nurses", "home nurse", "home nurses", "home health nurses", "home health nurse", "Home Care Services, Hospital-Based", "home health service", "home health services", "Health Plan Implementation", “Health Plan Implement*”, "Information Dissemination", “Information Disseminat*”, “Information Distribut*”, “Information Shar*”, “data shar*”, “multicomponent”, "Practice Guidelines as Topic", “implementation research”, "Guideline Adherence", “Policy Compliance”, “Protocol Compliance”, “barrier*”, "Health Knowledge, Attitudes, Practice", "Practice Patterns, Nurses”, “practice pattern*”.

Inclusion criteria were: systematic reviews or reports of empirical research performed in home care, research on the experienced barriers and facilitators for the implementation of non-pharmacological guidelines or health programs; research conducted in direct patient care; involvement of a multidisciplinary team (at least two professional home care providers). Research performed in institutional care, on pharmacological guidelines, health programs or guidelines concerning specific diseases and research concerning children were excluded.

One researcher (SV) screened the titles and abstracts of 4850 articles. The full texts of 56 potentially relevant research reports were then read by one researcher (SV), to see if they met the inclusion criteria. Further, the reference lists of the included studies were checked for additional relevant literature (SV). In total 16 articles were included. A summary of the findings was discussed with the research team and expert group of stakeholders.
