# Supplementary material for: Reducing the use of physical restraints in home care: development and feasibility testing of a multicomponent program to support the implementation of a guideline
Source: BMC Geriatr. 2021 Jan 25;21:77. doi: 10.1186/s12877-020-01946-5 (PMC7831193; doi:10.1186/s12877-020-01946-5)
Supplement: Supplementary file 2 — Additional file 2. Topic guide focus group interview IM step 1. [file 12877_2020_1946_MOESM2_ESM.docx]

**Additional file 2: Topic guide focus group interview IM step 1**

**Professional home care providers**

**General information about the aim of the study and the interview**

Despite the harmful effects of restraint use on patients, family caregivers and home care providers, it is still frequently used in home care. A validated evidence-based guideline was developed to reduce physical restraint use in home care. To get this guideline into practice, this study aims to systematically develop and evaluate a multicomponent program for the implementation of the guideline.

The aim of this focus group interview is to explore your experienced barriers and facilitators for the implementation of a guideline for physical restraint use.

Before we start, I want to discuss some general agreements:

- I would like to emphasize that everyone is free to speak.
- We are interested in both positive and negative comments.
- You do not have to agree with each other, but please show respect for each other's opinion.
- In order to understand everyone, I kindly ask you not to interrupt each other.
- You do not have to speak in the microphone, the tape recorder has a good range.
- The information obtained will be treated in strict confidence.
- I will ask questions, to get an answer to our research questions.
- We want to ask your approval to record this interview. We would like to emphasize that the recording will only be used for the purpose of this study. The audio recording will be deleted after completion.

# **Introduction round**

# **Opening questions**

1. What do you think is the meaning of physical restraints?
2. What do you think is the meaning of a guideline?
3. How is a guideline used within your organization?

**A researcher explains the content of the guideline for physical restraint use in home care.**

# **Questions**

1. What is your opinion of this guideline?
2. What do you expect from your organization so that you can use this guideline (policy, resources, time, support, training, cooperation)?
3. From your opinion, what are the benefits to use this guideline?
4. Why would you not use the guideline (organizational level, caregivers, patient level, collaboration, quality)?
5. What would you need to apply this guideline?

# **Closing question**

1. Would you like to mention or add anything that is important to you?
