# Supplementary material for: Reducing the use of physical restraints in home care: development and feasibility testing of a multicomponent program to support the implementation of a guideline
Source: BMC Geriatr. 2021 Jan 25;21:77. doi: 10.1186/s12877-020-01946-5 (PMC7831193; doi:10.1186/s12877-020-01946-5)
Supplement: Supplementary file 3 — Additional file 3. Methodology evaluation multicomponent program (knowledge test, online survey and two focus groups). [file 12877_2020_1946_MOESM3_ESM.docx]

**Additional file 3: Methodology evaluation multicomponent program (knowledge test, online survey and two focus groups)**

1. **Knowledge test**

The researchers developed a knowledge test based on the content of the guideline. The test had 0 as the minimum score and 31 as the maximum score. The test consisted of ten knowledge questions (e.g. ‘Is the use of physical restraints a solution for fall prevention?’ – yes/no), questions about the legislation of physical restraint use in Belgium (e.g. ‘Who can apply physical restraints when needed?’) and ethical statements (e.g. Is the following statement right or wrong? ‘Patient, informal caregiver and family need to be involved in the decision-making process regarding the use of restraints’). Descriptive statistics were used to analyze the data of the knowledge test.

1. **Online survey**

The online survey aimed to evaluate the different components of the developed multicomponent program (i.e. a website, social media, promo video, flyer, summary of the guideline, physical restraints checklist, tutorials and ambassadors for restraint-free home care). The ambassadors were asked to answer for each component the frequency of use and the degree of dissemination within the organization. In addition, the ambassadors needed to specify their level of agreement on a 5-point Likert scale (strongly disagree, disagree, unsure, agree, strongly agree) with statements on the clarity, the feasibility, the degree of support and the effect on specific determinants (e.g. awareness, knowledge, competencies, motivation, accessibility,…) of each component. Descriptive statistics were used to analyze the data of the online survey.

1. **Focus groups**

The focus groups followed a topic guide based on the findings of the knowledge test and the online survey. The topic guide consisted of general questions (e.g. ‘How do you experience the multicomponent-program?’, ‘Which components of the program do you evaluate more positively and least positively, why?’) and questions evaluating the process (e.g. ‘Which components have helped or supported you to implement the guideline?’, ‘What are the experienced barriers and facilitators to implement the guideline for physical restraint use in home care?’, ‘Are there components of the program that you adapted or used for other purposes within your organization?’) (additional file 4). The focus groups were recorded and notes were made during the interviews. One researcher (SV) listened to the recordings and made some additions to the notes. The contents of the written texts were thematically analyzed by two researchers independently (SV, KS). The two researchers discussed the main findings. Afterwards the findings were discussed within the research group.
