# Supplementary material for: Reducing the use of physical restraints in home care: development and feasibility testing of a multicomponent program to support the implementation of a guideline
Source: BMC Geriatr. 2021 Jan 25;21:77. doi: 10.1186/s12877-020-01946-5 (PMC7831193; doi:10.1186/s12877-020-01946-5)
Supplement: Supplementary file 4 — Additional file 4. Topic guide focus group interviews IM step 4. [file 12877_2020_1946_MOESM4_ESM.docx]

**Additional file 4: Topic guide focus group interviews IM step 4**

**Ambassadors for physical restraint-free home care**

# **General information about the aim of this research project and focus group interview**

The aim of this focus group interview is to determine whether the developed multicomponent program is applicable, feasible and workable. We also want to explore which components hindered or helped you to implement this guideline. We mainly want to investigate whether the program ensures that the guideline is taking into practice.

Before we start, I want to discuss some general agreements:

- I would like to emphasize that everyone is free to speak.
- We are interested in both positive and negative comments.
- You do not have to agree with each other, but please show respect for each other's opinion.
- In order to understand everyone, I kindly ask you not to interrupt each other.
- You do not have to speak in the microphone, the tape recorder has a good range.
- The information obtained will be treated in strict confidence.
- I will ask questions, to get an answer to our research questions.
- We want to ask your approval to record this interview. We would like to emphasize that the recording will only be used for the purpose of this study. The audio recording will be deleted after completion.

# **Opening questions**

1. What are your experiences with the multicomponent program?
2. Which components of the program do you experience most positively? How come?
3. Which components of the program do you experience least positively? How come?

# **Questions**

1. Which program components have helped or supported you to use the guideline in your daily practice?
2. What are the barriers and facilitators within your organization or work context to achieve a physical restraint-free home care?
3. Are there components of the program that you have used differently or adapted? If yes, what are the adjustments that you have made and why?

# **Closing question**

1. Would you like to mention or add anything that is important to you?
