# Supplementary material for: Reducing the use of physical restraints in home care: development and feasibility testing of a multicomponent program to support the implementation of a guideline
Source: BMC Geriatr. 2021 Jan 25;21:77. doi: 10.1186/s12877-020-01946-5 (PMC7831193; doi:10.1186/s12877-020-01946-5)
Supplement: Supplementary file 5 — Additional file 5. Results survey evaluation multicomponent program. [file 12877_2020_1946_MOESM5_ESM.docx]

**Additional file 5: Results survey evaluation multicomponent program**

| **Website** | | | | | |
| --- | --- | --- | --- | --- | --- |
|  | Never | Once | Monthly | Weekly | Daily |
| How many times did you visit the website? (n = 10) | 0 | 5 | 5 | 0 | 0 |
|  | Yes | No |  |  |  |
| Did you disseminate the website?  (n = 10) | 7 | 3 |  |  |  |
|  | Strongly disagree | Disagree | Agree | Strongly agree | No opinion |
| Is clear and logical. (n = 10) | 0 | 0 | 8 | 2 | 0 |
| Increases my awareness on the problem of physical restraint-use in home care. (n = 10) | 0 | 0 | 7 | 3 | 0 |
| Supports my daily practice. (n = 10) | 0 | 1 | 8 | 1 | 0 |
| **Social media (Facebook en Twitter)** | | | | | |
|  | Never | Once | Monthly | Weekly | Daily |
| How many times did you visit social media? (n = 10) | 5 | 4 | 1 | 0 | 0 |
|  | Yes | No |  |  |  |
| Did you disseminate social media? (n = 5) | 3 | 2 |  |  |  |
|  | Strongly disagree | Disagree | Agree | Strongly agree | No opinion |
| Increases my awareness on the problem of physical restraint use in home care.  (n = 5) | 0 | 0 | 3 | 1 | 1 |
| Supports my daily practice.  (n = 5) | 0 | 0 | 3 | 1 | 1 |
| Gives me information on the latest developments of the project.  (n = 5) | 0 | 0 | 3 | 1 | 1 |
| **Promo video** | | | | | |
|  | Yes | No |  |  |  |
| Did you see the promo video? (n = 10) | 9 | 1 |  |  |  |
|  | Yes | No |  |  |  |
| Did you use the promo video in your organization? (n = 9) | 6 | 3 |  |  |  |
|  | Strongly disagree | Disagree | Agree | Strongly agree | No opinion |
| Increases my awareness on the problem of physical restraint use in home care.  (n = 9) | 0 | 1 | 3 | 4 | 1 |
| Motivates me to work on a physical restraint-free home care. (n = 9) | 0 | 0 | 5 | 3 | 1 |
| Motivates my colleagues to work on a physical restraint-free home care. (n = 9) | 0 | 0 | 4 | 2 | 3 |
| **Flyer** | | | | | |
|  | Never | Once | Monthly | Weekly | Daily |
| How many times did you use the flyer? (n = 10) | 2 | 3 | 5 | 0 | 0 |
|  | Yes | No |  |  |  |
| Did you use the flyer in your organization? (n = 8) | 7 | 1 |  |  |  |
|  | Strongly disagree | Disagree | Agree | Strongly agree | No opinion |
| Increases the awareness of patient, informal caregiver and family on the use of physical restraints in home care. (n = 8) | 0 | 0 | 6 | 2 | 0 |
| Supports me to openly communicate restraint use with patient, informal caregiver and family. (n = 8) | 0 | 0 | 5 | 3 | 0 |
| It is feasible to communicate the use of physical restraints with the flyer. (n = 8) | 0 | 0 | 6 | 2 | 0 |
| **Summary of the practice guideline** | | | | | |
|  | Never | Once | Monthly | Weekly | Daily |
| How many times did you use the summary of the guideline? (n = 10) | 1 | 5 | 4 | 0 | 0 |
|  | Yes | No |  |  |  |
| Did you use the summary of the guideline within your organization? (n = 9) | 8 | 1 |  |  |  |
|  | Strongly disagree | Disagree | Agree | Strongly agree | No opinion |
| Increases my awareness on the problem of physical restraint use in home care.  (n = 9) | 0 | 1 | 6 | 2 | 0 |
| Supports me to cope with the demand to use physical restraints in home care. (n = 9) | 0 | 1 | 5 | 2 | 1 |
| Helps me to analyze the care context and to make a thoughtful decision on the use of physical restraints. (n =9 ) | 0 | 4 | 3 | 1 | 1 |
| Is feasible to use in my daily practice. (n = 9) | 0 | 4 | 4 | 0 | 1 |
| Is a handy tool. (n = 9) | 1 | 3 | 5 | 0 | 0 |
| Is clear. (n = 9) | 0 | 2 | 6 | 1 | 0 |
| **Physical restraints checklist** | | | | | |
|  | Yes | No |  |  |  |
| Did you see the physical restraints checklist? (n = 10) | 7 | 3 |  |  |  |
|  | Yes | No |  |  |  |
| Did you use the physical restraints checklist in your organization? (n = 7) | 5 | 2 |  |  |  |
|  | Strongly disagree | Disagree | Agree | Strongly agree | No opinion |
| Is accessible. (n = 7) | 0 | 0 | 5 | 2 | 0 |
| Supports me as an ambassador. (n = 7) | 0 | 0 | 5 | 2 | 0 |
| Helps me to document the care situation. (n = 7) | 0 | 0 | 4 | 2 | 1 |
| Helps me to document the decision-making process. (n = 7) | 0 | 0 | 4 | 2 | 1 |
| Is clear. (n = 7) | 0 | 0 | 5 | 2 | 0 |
| Is complete. (n = 7) | 0 | 2 | 5 | 0 | 0 |
| **Tutorial practice guideline** | | | | | |
|  | Yes | No |  |  |  |
| Did you see the tutorial? (n = 10) | 8 | 2 |  |  |  |
|  | Yes | No |  |  |  |
| Did you use the tutorial in your organization? (n = 8) | 6 | 2 |  |  |  |
|  | Strongly disagree | Disagree | Agree | Strongly agree | No opinion |
| Increases my awareness on the problem of physical restraint use in home care.  (n = 8) | 0 | 0 | 4 | 4 | 0 |
| Supports my daily practice.  (n = 8) | 0 | 1 | 3 | 4 | 0 |
| Motivates me to use the guideline. (n = 8) | 0 | 1 | 3 | 4 | 0 |
| Is worthwhile to see. (n = 8) | 0 | 0 | 4 | 4 | 0 |
| Is clear. (n = 8) | 0 | 0 | 4 | 4 | 0 |
| **Tutorial flowchart** | | | | | |
|  | Yes | No |  |  |  |
| Did you see the tutorial? (n = 10) | 5 | 5 |  |  |  |
|  | Yes | No |  |  |  |
| Did you use the tutorial in your organization? (n = 5) | 4 | 1 |  |  |  |
|  | Strongly disagree | Disagree | Agree | Strongly agree | No opinion |
| Supports my daily practice. (n = 5) | 0 | 0 | 3 | 2 | 0 |
| Motivates me to use the flowchart. (n = 5) | 0 | 0 | 3 | 2 | 0 |
| Explains how to use the flowchart. (n = 5) | 0 | 0 | 3 | 2 | 0 |
| Is worthwhile to see. (n = 5) | 0 | 0 | 3 | 2 | 0 |
| Is clear. (n = 5) | 0 | 0 | 3 | 2 | 0 |
| **Training to become an ambassador restraint-free home care** | | | | | |
|  | Strongly disagree | Disagree | Agree | Strongly agree | No opinion |
| Increases my awareness on the problem of physical restraint use in home care.  (n = 10) | 0 | 0 | 7 | 3 | 0 |
| Supports me as an ambassador. (n = 10) | 0 | 1 | 6 | 3 | 0 |
| Motivates me to use the guideline. (n = 10) | 0 | 2 | 6 | 2 | 0 |
| Ensures me to support other care providers to use the guideline. (n = 10) | 0 | 1 | 7 | 1 | 1 |
| Ensures me to gain trust of patient, informal caregiver and family. (n = 10) | 0 | 1 | 5 | 1 | 3 |
| Ensures me to give feedback to my colleagues. (n = 10) | 0 | 0 | 7 | 3 | 0 |
| Ensures me to cope with resistance of colleagues. (n = 10) | 0 | 0 | 7 | 2 | 1 |
| Ensures me to cope with resistance of patient, informal caregivers and family. (n = 10) | 0 | 1 | 6 | 0 | 3 |
| **Peer coaching 1** | | | | | |
|  | Strongly disagree | Disagree | Agree | Strongly agree | No opinion |
| Ensures me to gain insight in alternatives for restraint use. (n = 10) | 0 | 1 | 5 | 3 | 1 |
| Ensures me to understand the legislation regarding physical restraints in Belgium. (n = 10) | 0 | 0 | 6 | 3 | 1 |
| Motivates and inspires me as an ambassador. (n = 10) | 0 | 2 | 5 | 2 | 1 |
| Helped me as an ambassador. (n = 10) | 0 | 2 | 5 | 2 | 1 |
| Was relevant to share experiences. (n = 10) | 1 | 0 | 4 | 4 | 1 |
| **Peer coaching 2** | | | | | |
|  | Strongly disagree | Disagree | Agree | Strongly agree | No opinion |
| Supports me as an ambassador. (n = 10) | 0 | 1 | 6 | 1 | 2 |
| Ensures me to gain insight in alternatives for restraint use. (n = 10) | 0 | 1 | 5 | 2 | 2 |
| Ensures me to give feedback to my colleagues. (n = 10) | 0 | 1 | 6 | 1 | 2 |
| Ensures me to cope with resistance of colleagues. (n = 10) | 0 | 0 | 6 | 1 | 3 |
| Ensures me to cope with resistance of patient, informal caregivers and family. (n = 10) | 0 | 0 | 5 | 0 | 5 |
| Was relevant to share experiences. (n = 10) | 0 | 0 | 4 | 4 | 2 |
